# Validation of Novel spot blotch disease resistance alleles identified in unexplored wheat (*Triticum aestivum* L.) germplasm lines through KASP markers

**DOI:** 10.1186/s12870-022-04013-w

**Published:** 2022-12-29

**Authors:** Suneel Kumar, Anjan Kumar Pradhan, Uttam Kumar, Guriqbal Singh Dhillon, Satinder Kaur, Neeraj Budhlakoti, Dwijesh Chandra Mishra, Amit Kumar Singh, Rakesh Singh, Jyoti Kumari, Vikas V. Kumaran, Vinod Kumar Mishra, Pradeep Kumar Bhati, Saikat Das, Ramesh Chand, Kuldeep Singh, Sundeep Kumar

**Affiliations:** 1 ICAR-National Bureau of Plant Genetic Resources, New Delhi, India; 2 Borlaug Institute for South Asia, NASC Complex, DPS Marg, New Delhi, India; 3 Thapar Institute of Engineering & Technology, Patiala, India; 4 Punjab Agricultural University, Ludhiana, Punjab India; 5 ICAR-Indian Agricultural Statistics Research Institute, New Delhi, India; 6 ICAR-Indian Agricultural Research Institute, Regional Station, Wellington, India; 7 Banaras Hindu University, Uttar Pradesh, Varanasi, India; 8 Uttar Banga Krishi Vishwavidyalaya, Pundibari, Coochbehar India

**Keywords:** Triticum aestivum, Spot blotch, GWAS, MTAs, KASP marker, Validation, Marker-assisted selection (MAS)

## Abstract

**Background:**

During the last few decades, the diverse sources of resistance, several genes and QTLs for spot blotch resistance have been identified. However, a large set of germplasm lines are still unexplored that have the potential to develop highly resistant wheat cultivars for the target environments. Therefore, the identification of new sources of resistance to spot blotch is essential for breeding programmes to develop spot blotch resistant cultivars and sustain wheat production. The association mapping panel of 294 diverse bread wheat accessions was used to explore new sources of spot blotch disease resistance and to identify genomic regions using genome wide association analysis (GWAS). The genotypes were tested in replicated trials for spot blotch disease at three major hot spots in India (Varanasi in UP, Pusa in Bihar, and Cooch Behar in West Bengal). The area under the disease progress curve (AUDPC) was calculated to assess the level of resistance in each genotype.

**Results:**

A total of 19 highly and 76 moderately resistant lines were identified. Three accessions (EC664204, IC534306 and IC535188) were nearly immune to spot blotch disease. The genotyping of all accessions resulted in a total of 16,787 high-quality polymorphic SNPs. The GWAS was performed using a Compressed Mixed Linear Model (CMLM) and a Mixed Linear Model (MLM). A total of seven significant MTAs, common in both the models and consistent across the environment, were further validated to develop KASP markers. Four MTAs (AX-94710084, AX-94865722, AX-95135556, and AX-94529408) on three chromosomes (2AL, 2BL, and 3BL) have been successfully validated through the KASP marker.

**Conclusions:**

The new source of resistance was identified from unexplored germplasm lines. The genomic regions identified through GWAS were validated through KASP markers. The marker information and the highly resistant sources are valuable resources to rapidly develop immune or near immune wheat varieties.

**Supplementary Information:**

The online version contains supplementary material available at 10.1186/s12870-022-04013-w.

## Background

Wheat (*Triticum aestivum* L.) is known to be a primary food for more than one third of the world’s population (FAO, 2020). Concentrated efforts are being made to accelerate the genetic gain of wheat grain yields to meet the global food demand by the year 2050. Foliar blight (spot blotch) disease of wheat, caused by *Bipolaris sorokiniana,* is a major challenge in the way of achieving this projected global food demand. There are several shreds of evidence of significant yield loss, ranging from 18 to 50% in warm and humid regions of the world [1–3]. This may be as high as an 80% yield loss under severe infection [4]**.** Spot blotch disease is being reported to be spreading towards cool and conventional wheat-growing areas [5–7]**.** Due to global warming and a shift in environmental conditions, it is necessary to have a better understanding of the molecular genetics of spot blotch and to develop varieties or breeding material that are resistant to spot blotch in wheat. The spot blotch pathogen is highly variable, and its aggressiveness increases with time [8, 9]. The immunity to spot blotch disease in wheat is lacking. Therefore, assessment of disease levels due to the quantitative nature of resistance is difficult [10, 11]. It has been reported that the level of resistance to spot blotch is not up to the desired level in most of the high-yielding wheat varieties in India [4]. Keeping in view the erratic changes in the weather conditions, end user and grower driven demand, it is need of hour to explore the untapped resources from the gene banks and identify novel sources to develop better-performing, climate resilient and disease resistant cultivars [12]. Due to unavailability of germplasm with desired level of resistance, and reliable markers linked with the QTL(s) of significant effect on resistance to biotic and abiotic stresses, breeding wheat cultivars for higher yields and disease resistance has not made much headway [13, 14]. Consequently, a need for further exploration of resistance sources to combat spot blotch disease is suggested [15, 16].

Significant progress has been made in the identification of resistance genes and QTLs using germplasm lines and mapping populations in the last few decades [7, 17]. Several QTLs and genomic regions were identified using bi-parental mapping populations and a few using association mapping (AM) panels. The literature mining suggested that most of the wheat chromosomes were found to carry genes/QTLs or genomic regions that play a significant role in spot blotch disease resistance [7, 18]. Among these QTLs or genomic regions, 7D, 5B, 3B, and 4B were designated as genes *Sb1*, *Sb2*, *Sb3*, and *Sb4* and played significant roles in imparting resistance, respectively [19–22]. Three QTLs for spot blotch resistance (*QSb.cim-1B, QSb.cim-3B* and *QSb.cim-5A*) have also been identified by Zhu et al. [23]. Singh et al. [24] identified five additional diagnostic molecular markers (*Xgwm371, Xgwm425, Xgwm445, Xbarc59 and Xbarc232*) for spot blotch resistance, while Gurung et al. [2] identified eleven SNP markers linked to spot blotch resistance on chromosomes 1B, 5A, 5B, and 7B. Similarly, Singh et al. [25] identified a total of 13 QTLs with major and minor effects while Ayana et al. [26] identified six SNP markers for spot blotch resistance in winter wheat.

The genome wide association study (GWAS) has been used more often in the recent past to facilitate gene identification, cloning, functional characterization, and validation. Several complex traits have been successfully dissected using GWAS in wheat, such as wheat blast [27], fusarium head blight [28–30], multiple leaf spot diseases [2], the tan spot in European winter wheat [31], and leaf and stripe rust [27, 32, 33]. Resistance to spot blotch has also been successfully dissected in wheat by adopting the GWAS approach [34, 35]. More recently, Tomar et al. [36] identified eight new QTLs using the GWAS approach in advanced breeding lines tested under natural field conditions.

The advent of high-throughput SNP-based genotyping technologies followed by the faster development of **K**ompetitive **A**llele **S**pecific **P**CR (KASP, http://www.Lgcgenomics.com) has revolutionized wheat research with respect to the number of molecular markers mapped and linked with various traits of interest [37]. The KASP marker system is favored in wheat by researchers due to its low cost, locus specificity, and efficiency [38].

An attempt was made to identify highly resistant or near immune lines using the virgin germplasm (landraces, synthetic, indigenous, and exotic accession), popular wheat varieties released in India since 1960, and advanced breeding lines. The GWAS followed by marker validation was also done to identify new potential genomic regions associated with spot blotch disease resistance in these accessions.

## Results

### Phenotyping

The analysis of variance (ANOVA) revealed highly significant variations (*P* < 0.0001) for genotypes, environments, and genotype × environment interactions (Table S1). The Spot blotch disease severity (AUDPC) ranged from 102.51 in Env5 to 1297.63 in Env 4 (Table S2). The highest mean AUDPC value (562.35) was observed in Env 2, while the lowest (419.09) was in Env 5. However, the accessions of the association mapping panel were normally distributed for spot blotch AUDPC across five environments (Fig. S1). The heritability value based on AUDPC was estimated to be 0.82. The correlation coefficient (Table S3) among all the environments ranged from 0.68 (between Env1 and Env3) to 0.87 (between Env3 and Env5) (Table S3).

### Marker coverage, population structure and linkage disequilibrium.

The genotyping resulted in a total of 35,143 SNPs, which were further filtered for various quality parameters. The SNPs with less than 10% minor allele frequency, marker having > 5% missing values and also genotypes with > 5% individuals with missing SNP calls were excluded for further downstream analysis which ends up with a total of 16,787 polymorphic SNPs. All 16,787 SNPs were assigned to a physical location on the wheat genome by using the SNPs probes in BLAST search against wheat genome assembly i.e., IWGSC v2.0. The highest number of SNPs was assigned to the B sub-genome (6,372), followed by the A sub-genome (5,412) and the D sub-genome (5,003). The distribution of SNP markers across chromosomes ranged from 324 (Chromosome 4D) to 1,190 (Chromosome 2B) (Table S4 and Fig. S2).

**Fig. 1:**
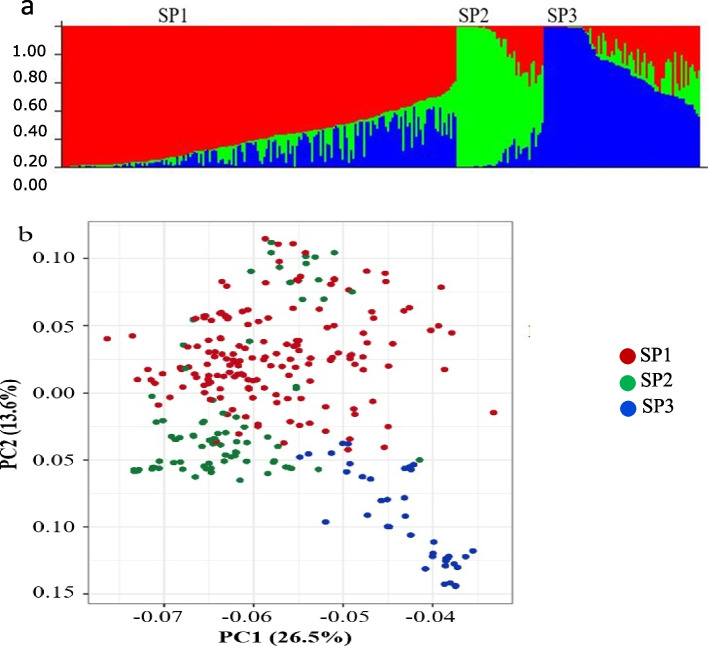
Population structure of spot blotch 294 genotypes. **a** bar plot, **b** PCA. Bar graphs for three subpopulations are indicated by different colours

The population structure analysis in the association mapping resulted in three sub-populations (SP, K = 3), that represent the distinct geographic collection of the germplasm lines. The subpopulation-1 (SP1) contains 179 genotypes (48–43% pure and 131–57% admixture) and includes wheat cultivars released in India and advanced breeding lines from the CIMMYT, Mexico. There were only 45 genotypes (19–43% pure and 26–57% admixture) falling into SP2 (Fig. 1a), dominated by the lines collected from the northern part of India. Subpopulation-3 consisted of 70 genotypes (24–34% pure and 46–66% admixture), which was the constitution of the genotypes across India except three lines from CIMMYT, Mexico. Pure and admixture types were considered based on membership proportion, i.e., ≥ 0.8 pure and the remaining as admixtures. SP1 was the largest among the three SPs, having 60% of the genotypes from the association mapping panel, followed by SP3 (23%) while SP2 was the smallest one (15%).

The principal component analysis (PCA) for the association mapping panel was also performed to further supplement the population structure analysis results obtained using the first three PCs. PC1 explained 26.5% of the genetic variance, whereas PC2 explained 13.6%. The PC scatter plot showed that the first and second PCs were composed largely of three SPs of accessions originating from different regions (Fig. 1b).

**Fig. 2:**
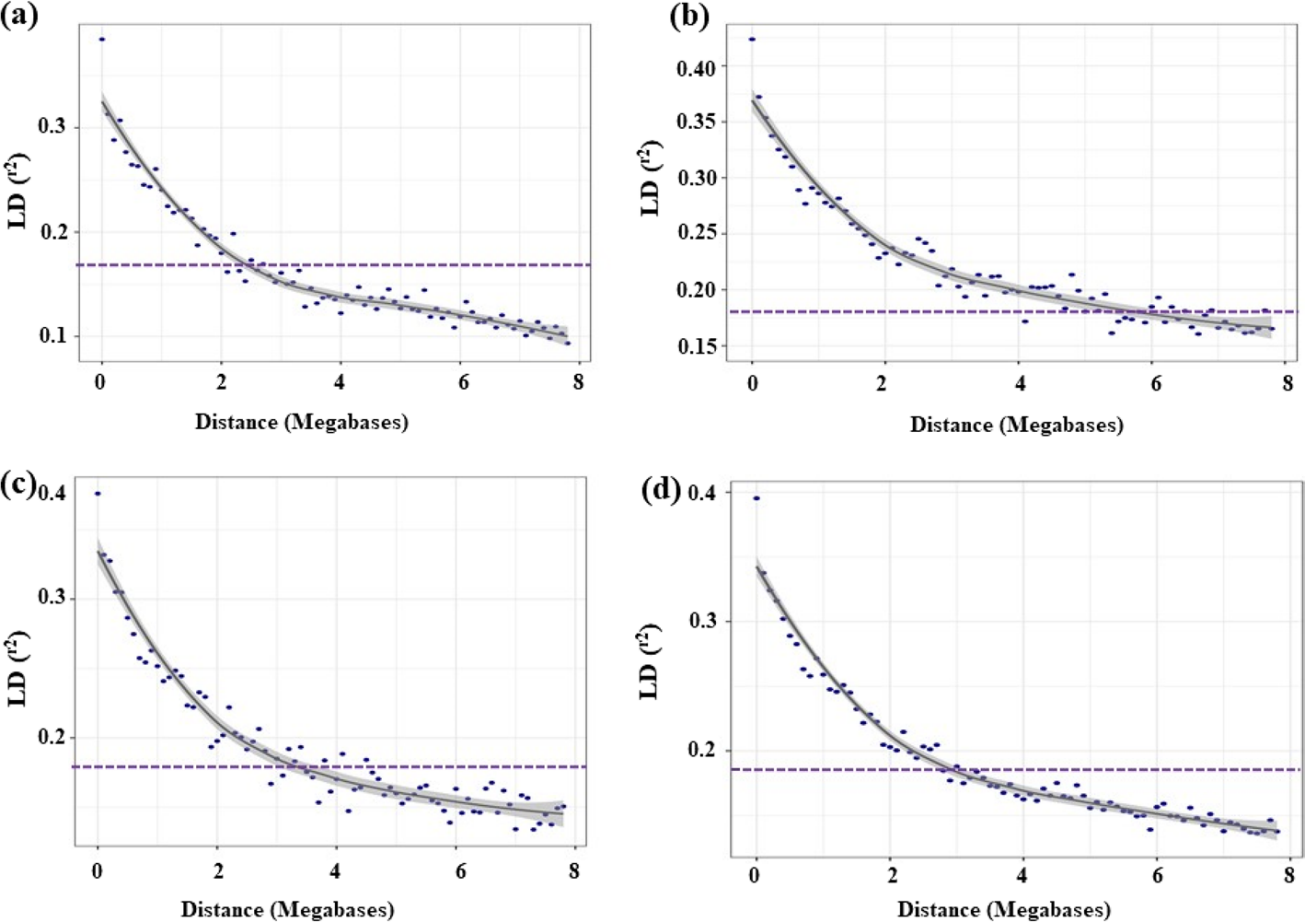
LD decay plot of association mapping panel **a** A-genome, **b** B-genome, **c** D-genome and **d** whole genome

A chromosome-wise linkage disequilibrium (LD) plot was also drawn for 16,787 SNP markers to investigate pair-wise linkage among markers. Individually, the average R^2^ of genome wide LD was 0.14 for sub-genome A, 0.15 for B, and 0.16 for D. SNP markers, with their assigned physical position on the map, were further used to estimate intra-chromosomal LD. The coefficient of regression (r^2^) for LD across 21 wheat chromosomes was minimum for chromosome 7D (0.087) and maximum LD was for chromosome 1D (0.367) (Table S4). Summery statistics of 16,787 markers indicating different levels of LD across different chromosomes within each sub-genome were also provided (Table S4). The fastest LD decay was observed for the A sub-genome, followed by B and D. In the A sub-genome, r^2^ value for the marker pair was reduced to 2.1 Mb as compared to 3.4 Mb in the D sub-genome and 4.6 Mb in B the sub-genomes. (Fig. 2 a-d).

### Genome wide association analysis

**Fig. 3:**
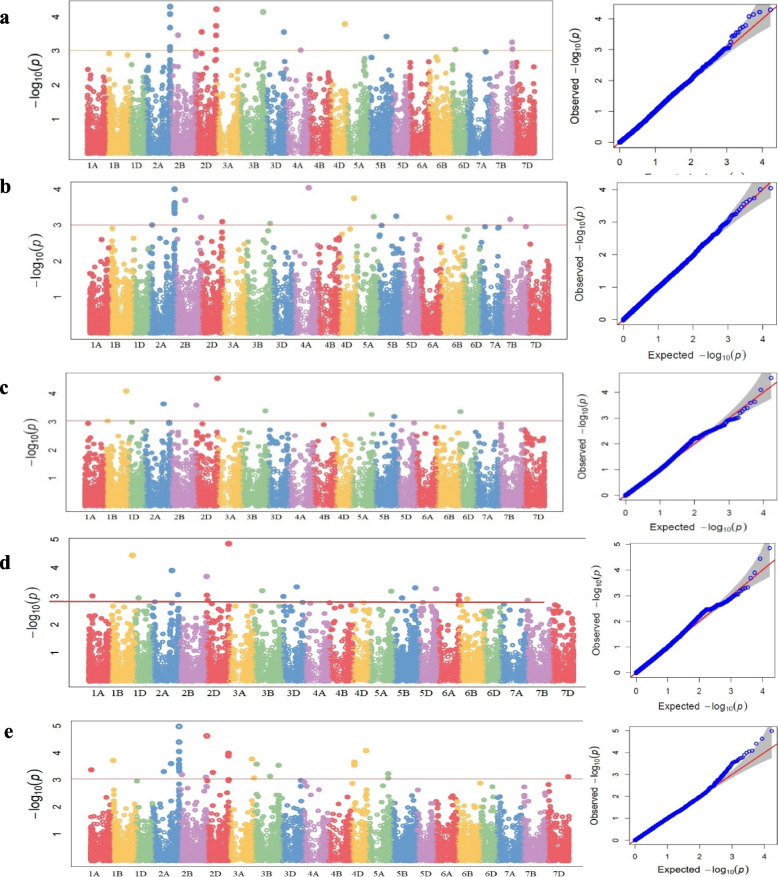
Manhattan plots and QQ plots for spot blotch AUDPC for **a** Env, **b** Env2, **c** Env3 **d** Env4 **e** Env5. The horizontal line represents the highly significant threshold at *P* < 0.001

A total of 240 marker trait associations were found in MLM, while 89 were obtained through CMLM by considering data from all five environments (Table S5 and Table S6). The threshold *P*-value to declare a significant marker trait association was considered as 1e-03 (Fig. 3 a-e). Further by considering the MTAs that are common to results of at least three environments, final number of significant markers were reduced to 21 for MLM and 10 for CMLM (Fig. 3 a-e).

These 21 MTAs identified through the MLM approach were distributed over 10 different chromosomes (2A, 3A, 6A, 1B, 2B, 3B, 2D, 3D, 4D, and 5D). It was observed that maximum number of MTAs was found on sub genome A (10), followed by B (6) and D (5) (Table S7). Considering the individual chromosomes, the highest number of eight MTAs were identified on chromosome 2A (AX-94495090, AX-94713668, AX-94685012, AX-94710084, AX-95135556, AX-94865722, AX-94445214, and AX-94889188), followed by chromosome 2B (AX-95217784, AX-94832247, and AX-94812374). Of the eight MTAs on chromosome 2A, six were located between 747 and 765 Mb of the genomic region where AX-95135556 was detected consistently in all environments except Env5. Similarly, the marker AX-94901587 located on 2D was consistently identified in four environments except Env2, while the marker AX-94794021 was present in all environments except Env5 on 3DL (Table S7).

The ten most consistent MTAs identified using the CMLM approach were located on chromosomes 2A, 2B, 2D, 3B, and 4D. The maximum number of five MTAs were identified on chromosome 2AL (AX-94749063, AX-94472501, AX-94710084, AX-95135556, and AX-94865722), followed by two on 2DL (AX-94901587 and AX-94559691) and one marker on each of 2BL (AX-95217784), 3B (AX-94529408), and 4DL (AX-94560557). The MTAs on chromosome 2AL were very small regions ranging from 763–765 Mb. Across five environments, the phenotypic variation for the most significant associations ranged from 30.1% (AX-94815439) to 32.0% (AX-94865722) (Table S8). The two MTAs (AX-94901587 and AX-94529408) were considered highly reliable as they were consistent in four environments, except Env2 and Env4 (Table S8). Finally, seven highly consistent MTAs common to both the MLM and CMLM approaches and also consistently present to the results of at least three environments, were further used for annotation and wet lab validation. These were distributed among five different chromosomes (2A, 2B, 2D, 3B, and 4D) (Table 1).

**Table 1:** Significant MTAs associated with the Spot blotch (AUDPC trait) in both models i.e., MLM and CMLM

Chr	Physical position (Mb)	TASSEL			GAPIT				
		Marker	*P*-Value	*R*^2^ (%)	*P*-value	FDR-P value	MAF	Allelic effect	*R*^2^ (%)
2AL	764.8	AX-94710084^a^	2.96E-04	6.4	2.09E-04	0.5022579	0.2293103	-72.324835	31.3
2AL	765.3	AX-94865722^a^	7.87E-05	7.6	5.11E-05	0.353617	0.2724138	68.810243	32.0
2AL	764.9	AX-95135556^a^	1.68E-04	6.4	8.43E-05	0.353617	0.3327586	57.005829	31.7
2BL	800.1	AX-95217784^a^	6.67E-05	7.3	6.05E-04	0.8003453	0.4507042	-58.429636	30.1
2DL	640.3	AX-94901587^a^	7.07E-04	5.8	1.86E-04	0.5022579	0.4948276	61.683789	31.3
3B-	719.8	AX-94529408^a^	4.54E-05	6.1	7.33E-05	0.353617	0.4965517	62.70801	31.8
4DL	442.2	AX-94560557^a^	5.28E-04	5.4	1.65E-04	0.5022579	0.3258621	55.154262	31.4

^a^Indicates present three or more environments. *FDR* False detection rate, *MAF* Minor Allele Frequency

### KASP Validation of identified MTAs

**Fig. 4:**
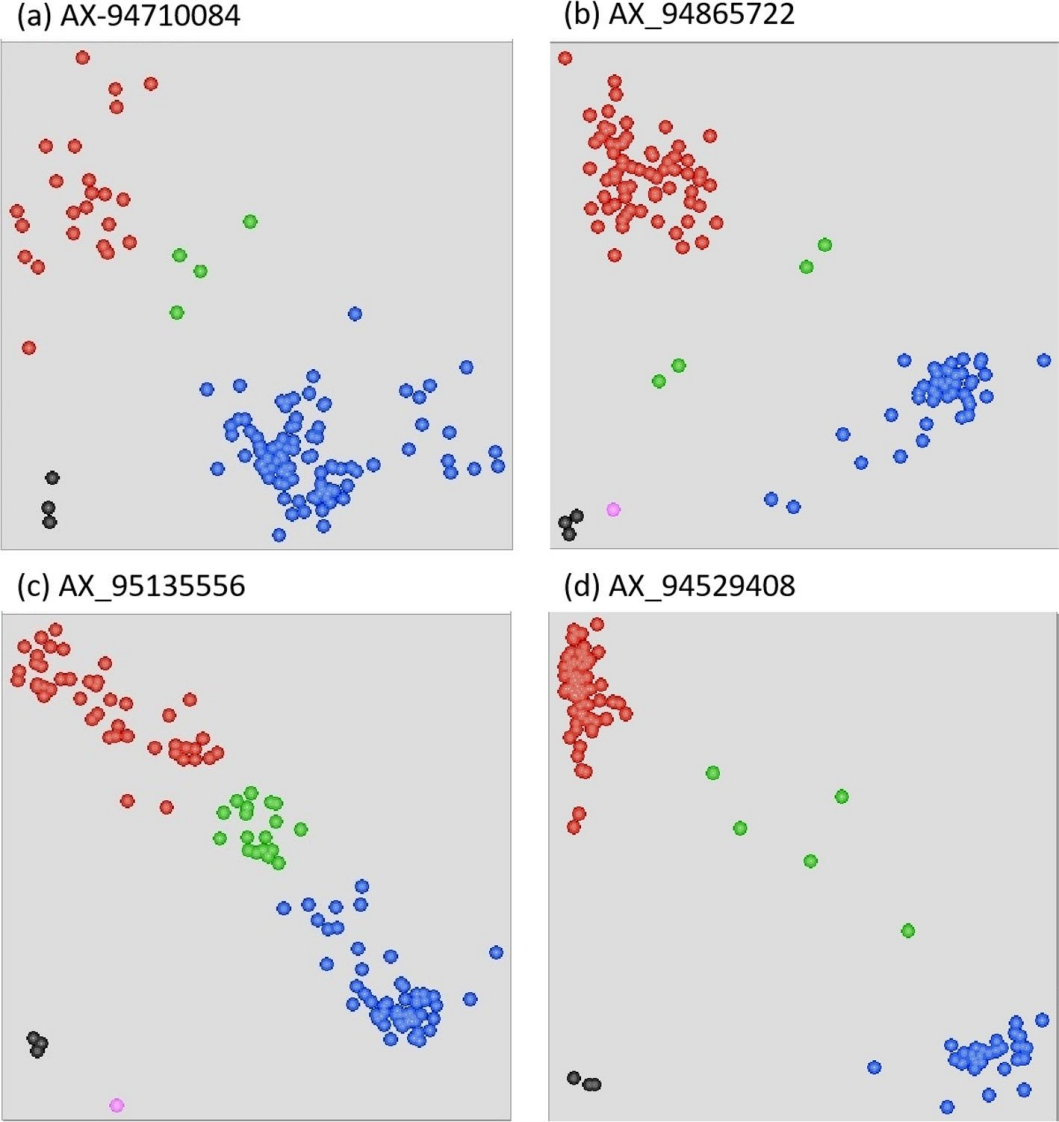
Genotypic evaluation of testing panel using KASP markers (**a**-**d**). The red and blue colour represents homozygous alleles, while the green colour represents heterozygous alleles. The black colour represents non-template control (NTC)

The KASP markers were designed for seven MTAs and amplified on an association testing panel. The sequences of these KASP markers are given in the supplementary Table S9. The KASP markers from four MTAs (AX-94710084, AX-94865722, AX-95135556, and AX-94529408) were polymorphic on the testing panel (Fig. 4 and Fig S3). These four markers were then amplified on a testing panel of 120 extremely highly resistant and susceptible germplasm lines as well as on the whole association panel. The alternate alleles of the respective markers had a significant effect on resistance across multiple environments (Fig. S4 and Fig S5). However, three of four SNP markers (AX-94710084, AX-94865722, and AX-95135556) from chromosome 2AL showed a high level of significance for MTA across environments. During validation, we identified the specific alleles contributing to resistance. Allele G of markers AX-94710084 and AX-94865722 (*p*-value < 0.001) and allele C of marker AX-95135556 (*p*-value < 0.05) were associated with resistance in all the environments. The alleles were present within the 1 Mb region of chromosome 2A (764.8–765.3). The allele T of marker AX-94529408 from chromosome 3B was associated with spot blotch disease resistance with a *p*-value of < 0.1.

## Discussion

The importance of wheat as a staple food all around the world is well known. In recent years, wheat has been suffering from a number of biotic and abiotic stresses globally, which necessitates the development of high yielding, climate resilient wheat cultivars to feed the growing population [39, 40]. Spot blotch disease of wheat is a major biotic threat to the wheat crop, with severe infection causing up to 80% yield losses [41]. Identification of new sources of resistance to spot blotch disease is a prerequisite for wheat breeding program to develop spot blotch resistant varieties [42]. With the objective of identifying the new sources of resistance in unexplored germplasm and new genomic regions for resistance to spot blotch, we used a diverse set of 294 wheat genotypes, including landraces, indigenous and exotic germplasm lines, advanced breeding lines, and popular wheat varieties of India. Landraces are well known as a repository for gene pools, sources of genetic variability, and a high potential for adaptation to specific environmental conditions [43–45], whereas other germplasm represents genetic variations used in various breeding programmes as sources of variations for multiple traits. This panel was expected to possess novel alleles for multiple traits, including spot blotch, and was thus selected for the present study.

Spot blotch disease resistance was tested by exposing the association mapping panel to three different agro-climatic zones of India, i.e., Varanasi, Uttar Pradesh (25.2677° N, 82.9913° E), PUSA, Bihar (25°57′22.8"N, 85°40′13.1"E) and West Bengal (26.3452° N, 89.4482° E) which are well known hotspots for this disease [46]. Although these locations are well known hot spots for the spot blotch, we created the artificial epiphytotic conditions using a highly aggressive isolate of *Bipolaris sorokiniana* (NABM MAT1; NCBIJN128877, BHU, Varanasi, India) [41] to reduce the chances of disease escape by any accession. However, there is a recent report where significant variation for spot blotch resistance was observed and QTLs were mapped in advanced breeding lines of wheat under natural conditions at PUSA, Bihar [36].

Spot blotch disease severity was recorded as a visualised percentage of the infected leaf area on a 0 -100 scale at three different growth stages following Kumar et al. [20]. We used AUDPC in place of individual scoring or average severity, which is considered to represent a more reliable means of assessing spot blotch resistance [47]. Our results were in agreement with previous findings those of Kumar et al. [20] and Tomar et al. [36], where percent disease severity at GS77 mainly reflected the AUDPC. The nearly continuous distribution of AUDPC in the association mapping panel confirms the significant genetic variability, while moderately high heritability suggests non-significant genotype × environment interactions. Earlier findings suggested that spot blotch resistance is controlled by two or more genes [20, 36, 48–52] or QTLs with minor to major effects [25, 26, 53]. We observed 36 and 55 genotypes with AUDPC values lower than the resistant checks ‘Yangmai #6’ and ‘Chirya #3', respectively. Of these highly resistant lines, IC290156 is an indigenous landrace, and possibly not used in any of the breeding program in India. This landrace has been reported to be resistant to leaf rust and stem rust as well (unpublished data).

The distribution and density of polymorphic markers reflect the overall genetic richness and diversity of the wheat genome. The D genome had the least average marker density, ranging from 0.64–1.85 SNPs/Mb as compared to the A (0.69–1.50) and B (0.9–1.54) genomes. Total markers used in the association mapping panel from the D genome were smaller (29.8.2%) compared to A (32.2%) and B (37.9%), suggesting relatively lower genetic diversity and a lower level of effective recombination in the D genome than observed in previous studies [54, 55].

Population structure or classification is one of the major limitations of GWAS. Differences in the allele frequencies among the subpopulation can lead to falsely detected MTAs or QTLs [56, 57]. In the current study, STRUCTURE program-based approaches determined three subpopulations in the association mapping panel. We eliminated the influence of ascertainment bias in the characterization of population structure and in assigning population structure and genetic relatedness as cofactors in GWAS. Structure analysis resulted in the identification of three subpopulations that varied in their allele frequencies (Fig. 2a).

PCA results also confirmed our results by dividing the association mapping panel into the same three different subpopulations. Not surprisingly, population structure represents the geographic distribution of germplasm lines, and a significant level of admixture represents the sharing of germplasm in different breeding programs across the globe. Most of the genotypes within the SP1 are wheat cultivars released in India and advanced breeding lines (71% from Indian wheat breeding programs and 29% from the CIMMYT, Mexico) that show the interdependence of both the breeding programs (India and CIMMYT). The SP2 was dominated by the local germplasm/ Landrace collection from the northern part of India, one of the major wheat producing parts of the country and reflects that these local germplasm or landraces are not included in any of the breeding program. While SP3 was the mix-up of the genotypes across India, except for three lines from CIMMYT, Mexico.

The distance between SNP and spot blotch resistance has been addressed in physical distances of mega-base (Mb) pairs rather than the genetic distance in centiMorgans, which has been used in some previous studies [58–60]. Our results demonstrated that the D genome had a high LD decay distance (4.6 Mb) compared to the A and B genomes (Fig. 2). However, a lower rate of average LD decay (higher distance) was observed in European hexaploid wheat (23 cM) [61] and US Elite hard red winter wheat (10 cM) at r2 > 0.1 [62]. The LD decay analysis in bread wheat for spot blotch showed variation among genomes and within the genome itself, indicating variability in recombination hot spots, differences in selection pressure imposed on alleles of wheat genomes, and evidence of recombination events in past breeding history.

GWAS enables high resolution mapping of traits, it might also reveal false positive associations if the confounding factors like population structure and genetic relatedness among the genotypes of the association panel are not accounted for. Therefore, in our analysis, a CMLM based method which accounts for both of these factors was used, which is suggested to be a more pragmatic approach in MTA [63]. To identify the most reliable and consistent markers, we selected SNPs common to both, MLM and CMLM approaches and also consistently present to the results of at least three environments. Therefore, a total of seven MTAs distributed across different chromosomal regions, i.e., Chromosome 2A, 2B, 2D, 3B, and 4D were identified (Table 1).

Several genomic regions for resistance to spot blotch have been reported on different chromosomes in earlier studies [2, 19, 21, 23, 25, 26, 34, 36, 49, 50, 52, 53, 64–69]. Only four major QTLs are designated as Sb1 on Chromosome 7D [19], Sb2 on Chromosome 5B [20], *Sb3* on Chromosome 3B [21], and Sb4 on Chromosome 4BL [22] are well described. We also detected seven consistent MTAs on different chromosomal regions, i.e., 2AL, 2BL, 2DL, 3BL, and 4DL, which were detected by both the models in a minimum of three environments (Table 1). We also tried to compare the location of identified QTLs in the current study with that of the previous studies. The exact comparison of such QTLs across different studies was difficult due to the difference in the number of markers, mapping populations, and genotyping techniques (SSRs or SNPs) used in these studies. Therefore, such QTLs were compared based on the mapped position on the chromosome (short arm or long arm) and were used in our analysis. The details of the previously identified QTLs are provided in the supplementary (Fig. S6).

The chromosome 2A seems to be a gene rich region where, out of seven, three MTAs (*AX-94710084*, *AX-94865722,* and *AX-95135556*) were located on the long arm. The marker *AX-94710084* located at 764.8 Mb explained up to 31.3% of the phenotypic variation for AUDPC. This marker belongs to the same chromosomal region where QTLs have been mapped in earlier studies (*S2A_704446408* by Tomar et al. [36] and SSR marker *Xgwm445* at 683 Mb by Kumar et al. [50]). Similarly, markers, *AX-94865722* and *AX-95135556,* located close to *S2A_703427639* and *S2A_703391992* at 765.3 and 764.9 Mb, explained 32.0% and 31.7% of phenotypic variation, respectively [36]. The study on spot blotch resistance in Afghan wheat germplasm also revealed a SNP of *1,215,338* markers on 2BL at 800 Mb, which is close to the *AX-95217784* (800.1 Mb) identified in our study [70]. The same was true in the case of marker *AX-94529408* on chromosome 3BL (719.8 Mb) which is located in the vicinity of SNP *7,354,241* [70]. The marker *AX-94901587* on 2DL (640 Mb) was mapped close to *S2D_389463371* [71] and *QSb.sdsu-2D.1* (Kukri_c31121_1460) [26]. Out of seven, one MTA (*AX-94560557*) was placed at 442.2 Mb on chromosome 4DL. Literature mining did not result in any reports of mapping in this region. Therefore, this region might have a novel allele for spot blotch resistance since unexplored germplasm was used in the current study.

The Bonferroni Holm correction is used to reduce false positives in previous studies [28–30, 32, 33, 63, 72]. However, there are reports that shown significantly large false negatives with Bonferroni Holm correction in complex and large genomes such as wheat [73]. To reduce the changes of false positive, we considered an MTA as stable and positive only if detected in at least 3 of the 5 environments. Moreover, since the phenotyping was performed in three geographically distinct regions, the effect of such variation on expression of a highly quantitative trait like spot blotch resistance is quite large. The effect of the lower heritability on selection of lower p-value cut-off has already been established in various studies showing overcorrection/adjustment of p-value in multiple-comparison correction when number of tests increases to thousand folds. Additionally, to the false positives were eliminated using two different algorithms; MLM and CMLM accounting for the Q-matrix (pedigree) and K-matrix (kinship).

It is interesting that three genotypes, namely, EC664204, IC534306 and IC535188, carry favourable alleles for spot blotch as well as for stripe rust and leaf rust resistance [74]. Therefore, this study provides opportunities to develop multi disease resistant cultivars. Similarly, the previous report by Kumar et al. [75] identified IC416188 as a genotype that contains a favourable allele for heat tolerance. This genotype contains favourable alleles for spot blotch as well. Therefore, such genotypes may provide a basis to combine multiple traits in an adapted genotype to develop climate resilient varieties [76].

All seven markers found in MTA were further used for KASP validation. The four MTAs (AX-94710084, AX-94865722, AX-95135556, and AX-94529408) have been successfully validated (Fig**.** S4) and it is observed that the genomic region on chromosome 2A (~ 765 Mb) was most significant for spot blotch resistance in the association mapping panel. During the validation of KASP markers a subset of highly resistant and highly susceptible lines was used including landraces, synthetic, indigenous, and exotic accessions. The theoretical heterozygosity does reduce to < 1% in inbred lines, while observed heterozygosity in landraces, varieties or other inbred lines in wheat has been reported to be up to 20% in various studies [54, 77]. Further, in various plant species residual heterozygosity have been found to be associated with higher gene densities and recombination rates underlining the complex genetic architecture [78, 79].

Further, these diagnostic markers could be utilized in future marker assisted breeding programs to rapidly select the genotypes carrying spot blotch disease resistant alleles.

### Identification of potential candidate genes

Candidates genes identified using e!Ensemble Plants (http://plants.ensemble.org/index.html) against the reference genome of *Triticum aestivum* and results of same are presented in Table 2. Further a detailed ontology (biological process, molecular function, cellular component) and pathway analysis of identified candidate genes has also been performed (Supplementary Table S10a-S10f & Fig. S7).

**Table 2:** Candidate genes identified in significantly associated SNPs with spot blotch resistance

SNP	Chromosome	Start (pos)	End (pos)	Gene	Description
AX-95135556	2AL	764,819,041	764,821,652	TraesCS2A02G564900	Leucine-rich repeat domain superfamily
AX-94710084	2AL	764,783,606	764,792,014	TraesCS2A02G564800	Leucine-rich repeat domain superfamily
AX-95217784	2BL	800,119,910	800,125,814	TraesCS2B02G628800	Uncharacterized protein At4g26450-like
AX-94901587	2DL	640,297,481	640,299,386	TraesCS2D02G577000	Protein kinase-like domain superfamily
AX-94529408	3B-	719,773,163	719,779,367	TraesCS3B02G471100	DEK, C-terminal
AX-94560557	4DL	442,164,847	442,169,345	TraesCS4D02G271400	PLAC8 motif-containing protein
AX-94865722	2AL	765,138,703	765,141,326	TraesCS2A02G565700	Cytochrome P450 superfamily

All seven markers were annotated for their underlying putative candidate genes (Table 2). A nucleotide binding site -leucine-rich-repeats (NBS-LRR) transporter gene (TraesCS2A02G564900) was found in the genomic region associated with KASP marker *AX-95135556* on the long arm of chromosome 2A that may be responsible for maintaining pathogen recognition and resistance. Similarly, another gene (TraesCS2A02G564800) was localized in the same genomic region associated with KASP marker, *AX-94710084*. A protein kinase-like domain superfamily resistance gene (TraesCS2D02G577000) was found in the genomic region associated with the marker AX*-94901587* located on long arm of chromosome 2DL. Earlier studies also reported similar results like involvement of diverse classes of genes encoding for Leucine-rich repeat receptor-like protein kinase family, which plays an important role in disease resistance to spot blotch [26, 36].

## Conclusion

The present study revealed one novel genomic region for spot blotch resistance on chromosome 4DL while confirming previously reported QTL on chromosomes 2A, 2B, 2D, and 3B. The KASP markers validated in the present study will be useful in SNP assisted pyramiding of multiple QTLs to attain a high level of resistance against spot blotch disease resistance. Additionally, the genotypes with multiple stress tolerance (biotic and abiotic) could be a winning combination to combat the battle against various biotic and abiotic stresses in breeding for the development of high yielding and climate-resilient wheat cultivars.

## Methods

### Plant Materials

A set of 239 wheat accessions (landraces, synthetic, indigenous, and exotic accessions) and popular wheat varieties released in India in different regions were obtained from the Indian Council of Agricultural Research – National Bureau of Plant Genetic Resources (ICAR-NBPGR), New Delhi, while 55 advanced breeding lines were obtained from the global wheat program of CIMMYT, Mexico.

All 294 germplasm accessions (called as association mapping panel, Table S11) were multiplied at ICAR-Indian Agricultural Research Institute (IARI), Regional Station, Wellington, Tamil Nadu. The single ear head from each accession was selected and covered just before the anthesis to maintain genetic purity through self-pollination. The well-known resistant (‘Yangmai #6’ and ‘Chirya #3’), and susceptible (Sonalika) checks were used for the disease comparison [49, 50].

### Field experiment for disease evaluation

The association mapping panel was evaluated for spot blotch during two consecutive crop seasons at the agriculture research farm of Banaras Hindu University (BHU), Varanasi, Uttar Pradesh (2017–18 as Env1 and 2018–19 as Env2), Borlaug Institute for South Asia (BISA) PUSA, Bihar (2017–18 as Env3 and 2018–19 as Env4) and Uttar Banga Krishi Viswavidyalaya (UBKVV), West Bengal (2017–18 as Env5). The germplasm lines were planted in Augmented block design in two replications at each location in each season, where each genotype was planted as four rows of one metre. The pure culture of the most aggressive isolate of *Bipolaris sorokiniana* identified at BHU Varanasi (NABM MAT1; NCBIJN128877, BHU, Varanasi, India) [41] was used to create artificial epiphytotic in the field. The isolate was multiplied by culturing on sorghum grains as described by Chand et al. [5]. An aqueous spore suspension with a concentration of 10^4^ spores ml^−1^ was sprayed on the plants using the hand-held sprayer during the evening hours at two different growth stages (GS) namely, time of early heading (GS53) and heading complete (GS57) [80]. The plots were irrigated immediately after inoculation and sufficient moisture was maintained during the disease development to get a good level of infection.

### Disease scoring

The disease response was visually recorded following [49] at GS63 (early flowering stage), GS69 (flowering complete), and GS77 (late milk stage) on a 0 to 100 scale, where 0 is immune and 100 is completely susceptible. The area under disease progress curve (AUDPC) value was calculated based on the disease severities recorded at the three growth stages using the formula given below [81, 82] as:

$$\mathrm{AUDPC}=\sum_{\mathrm i=1}^{\mathrm n-1}\left[\left\{\frac{\left({\mathrm Y}_{\mathrm i}+{\mathrm Y}_{\left(\mathrm i+1\right)}\right)}2\right\}\times\left({\mathrm t}_{\left(\mathrm i+1\right)}-{\mathrm t}_{\mathrm i}\right)\right]$$

where Y_i_ represents the disease level at time t_i_; t _(i+1)_-t_i_ is the number of days between consecutive disease scoring, and n is the number of scorings.

### Statistical analysis

Analysis of variance (ANOVA) was performed using SAS v9.3 (software) to further estimate associated variance components like genotype, environment, and their interaction for spot blotch disease severity. Heritability was also estimated using the restricted maximum likelihood (REML) method (https://rdrr.io/cran/qle/man/reml.html; accessed on Oct 05, 2021). A mean AUDPC value of each genotype was also calculated. The phenotypic data distribution was visualized using the histogram.

### Genotyping of GWAS Panel and SNP variant calling

The genomic DNA was isolated using the CTAB method of Doyle et al. [83] from approximately 300 mg leaf tissue of 15-days old seedlings. The genotyping of all accessions was performed using 35 k Axiom® Wheat Breeders Array according to the procedure described by Affymetrix (Axiom® 2. 0 Assay for 294 samples, P/N 703,154 Rev. 2). The SNP markers with minor allele frequency (MAF < 10%) across all genotypes were excluded from the analysis.

### Population structure, LD, and Kinship analysis

Model-based clustering approach implemented in STRUCTURE v2.3.4 [84] was used to determine the population structure. The expected number of sub-populations was chosen from the principal coordinates (PCO) plot, i.e., K *vs*. Delta K, where the rate of change in the log probability between consecutive K values was the highest number of sub-populations. The best fit number of clusters was estimated following the approach of Evanno et al. [85] implemented in a web based tool ‘Structure Harvester’ (http://taylor0.biology.ucla.edu/structureHarvester/; accessed on Oct 05, 2021) [86]. For each specified K, ten iterations of STRUCTURE were conducted with additional parameters including 10,000 burn-in period length and 20,000 Markov Chain Monte Carlo (MCMC) iterations after burn-in [86]. The optimum number of subpopulations (K) was estimated using adhoc statistics ΔK [85]. The principal components (PC) and marker kinship matrix were also obtained in GAPIT using the R package.

Linkage disequilibrium (LD) between each pair of markers was estimated using a utility in TASSEL v5 [87]. The background LD in the wheat AM panel was calculated to identify the critical distance for LD decay. LD decay distance across the sub-genomes and whole genome was estimated by plotting the scatter plot of LD *r*^*2*^ values between marker pairs and the inter-SNP physical distance. The critical r^2^ value that shows the area beyond which LD is due to true physical linkage was determined using the 95^th^ percentile of the square root of the transformed r^2^ data of unlinked markers [88]. Further, the intersection of the LD decay curve was observed at r^2^ = 0.176 and the same was used as a background threshold for linkage disequilibrium.

### Genome-wide association analysis

To identify SNP loci associated with spot blotch resistance, GWAS was conducted using 16,787 polymorphic SNP markers and AUDPC values. Marker-Trait Associations (MTAs) were estimated using two approaches, namely, Compressed Mixed Linear Model (CMLM) implemented in the GAPIT (Genomic Association and Prediction Integrated Tool) of R v3.5.3 package and Mixed Linear Model (MLM) implemented in TASSELv5. We also accounted for additional information like population structure (Q) through a PCA and relationships among individuals through a kinship (K) matrix, during the model fitting. Association analyses were conducted for each location across all environments, and the results were presented accordingly. The significance threshold value (P-value) for MTAs was set as < 0.001. Therefore, all MTAs below this threshold were considered significant. Further, to find candidate genes, each significantly associated MTA in relation to spot blotch were BLAST against wheat genome using plant e!Ensemble (https://plants.ensembl.org/index.html). The genes found in the overlapping region within 1 Mb upstream and downstream of the matched regions were considered as candidate genes and their sequence were extracted [89]. Further, gene ontology (GO) analysis of identified candidate genes were performed using BLAST2GO v5. [90]

#### Validation of Identified MTAs

The validation of the MTAs was done by designing Kompetitive Allele Specific Polymerase Chain Reaction (KASP) markers from the significant SNPs. Based on the physical position of significant SNPs, a 100 bp sequence from either side of SNPs was retrieved from the Chinese Spring reference genome RefSeqv1.0. The PCR based markers were designed using bio-polyploid-tools v0.9.7 [91]. The KASP markers were applied to a validation set of 120 new germplasm lines (Table S11) as well as to a subset of the association mapping panel 0f 294 genotypes used for GWAS (Table S12). The validation set was phenotyped at two locations (BHU, Varanasi in Uttar Pradesh and BISA, Pusa in Bihar) under artificial epiphytotic conditions as mentioned in the material and methods section. The validation set was divided in two groups (resistant and susceptible). Out of 120 genotypes (Table S11), 56 were resistant (0–30%), while 64 genotypes were characterized as susceptible (60–90%). Using the homozygous alleles of the genotypic data from KASP markers, the alternate alleles were tested for a significant difference using the Kruskal test [92, 93]. The list of highly resistant and moderately resistant lines based on phenotypic evaluation are listed in Table S13 and S14 respectively.

## Supplementary Information

**Additional file 1.**

**Additional file 2.**

**Additional file 3.**

**Additional file 4.**

## Data Availability

All data generated or analysed during this study are included in this published article and its supplementary information file.

## Abbreviations

GWAS: Genome wide association analysis
AUDPC: Area under the disease progress curve
CMLM: Compressed mixed linear model
MLM: Mixed linear model
MTAs: Marker trait associations
KASP: Kompetitive allele specific PCR
AM: Association mapping
ANOVA: Analysis of variance
SP: Sub population
PCA: Principal component analysis
LD: Linkage disequilibrium
GS: Growth stages
