# Supplementary material for: Validation of Novel spot blotch disease resistance alleles identified in unexplored wheat (Triticum aestivum L.) germplasm lines through KASP markers
Source: BMC Plant Biol. 2022 Dec 29;22:618. doi: 10.1186/s12870-022-04013-w (PMC9798658; doi:10.1186/s12870-022-04013-w)
Supplement: Supplementary file 1 — Additional file 1. [file 12870_2022_4013_MOESM1_ESM.docx]

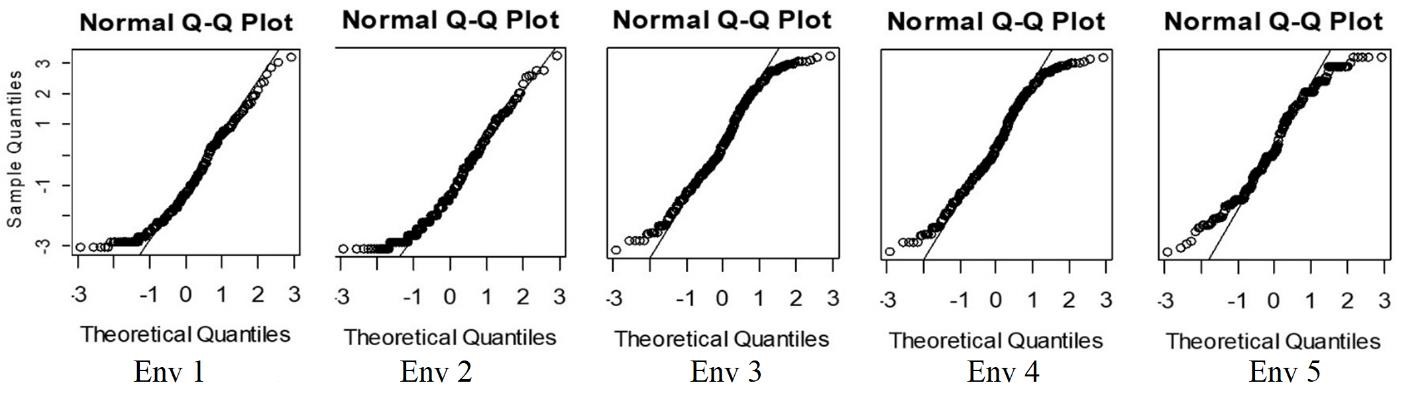


**Fig. S1** QQ plot of Spot blotch AUDPC across 5 different environments (Env1, Env2, Env3, Env4, Env5)


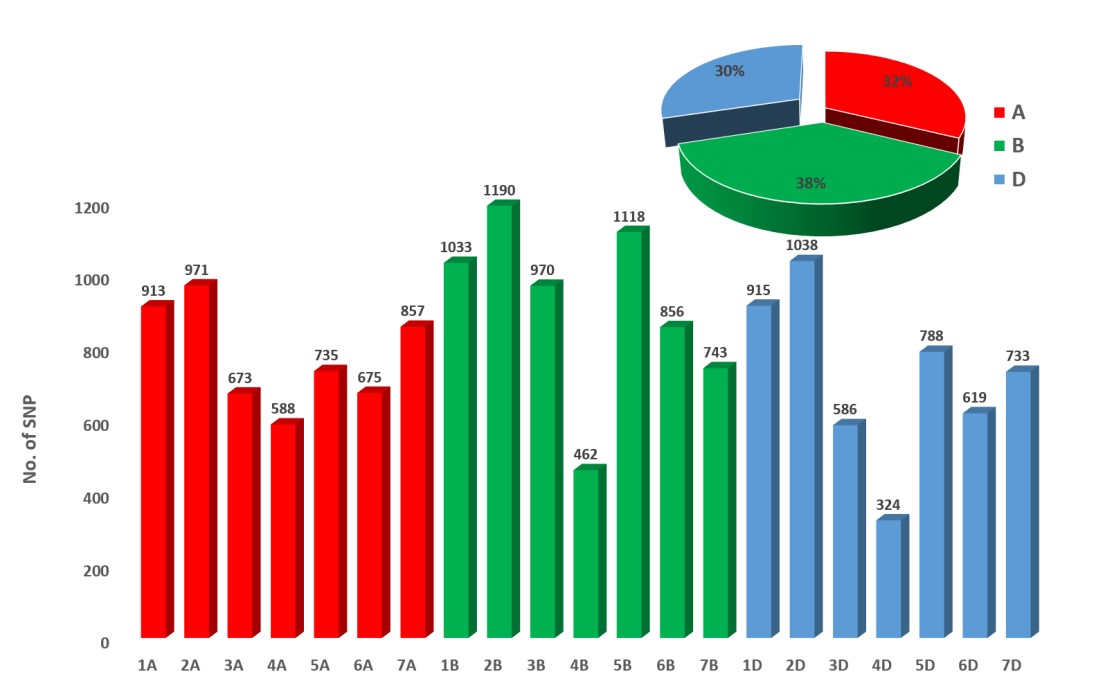


**Fig. S2** Distribution of 16,787 SNPs in 21chromosomes, identified LD in 294 bread wheat genotypes


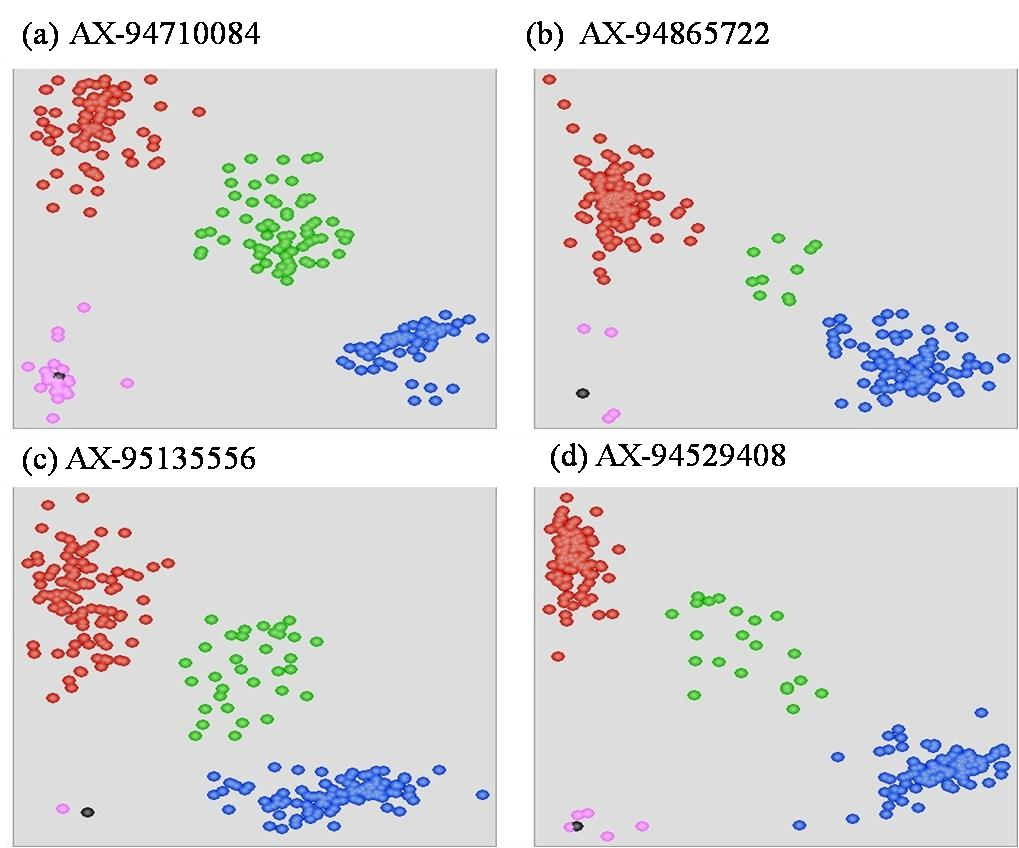


**Fig. S3** Genotypic evaluation of testing panel using KASP markers (a-d). The red and blue colour represents homozygous alleles, while the green colour represents heterozygous alleles. The pink colour represents not amplified samples, and the black colour represents non-template control (NTC)

| 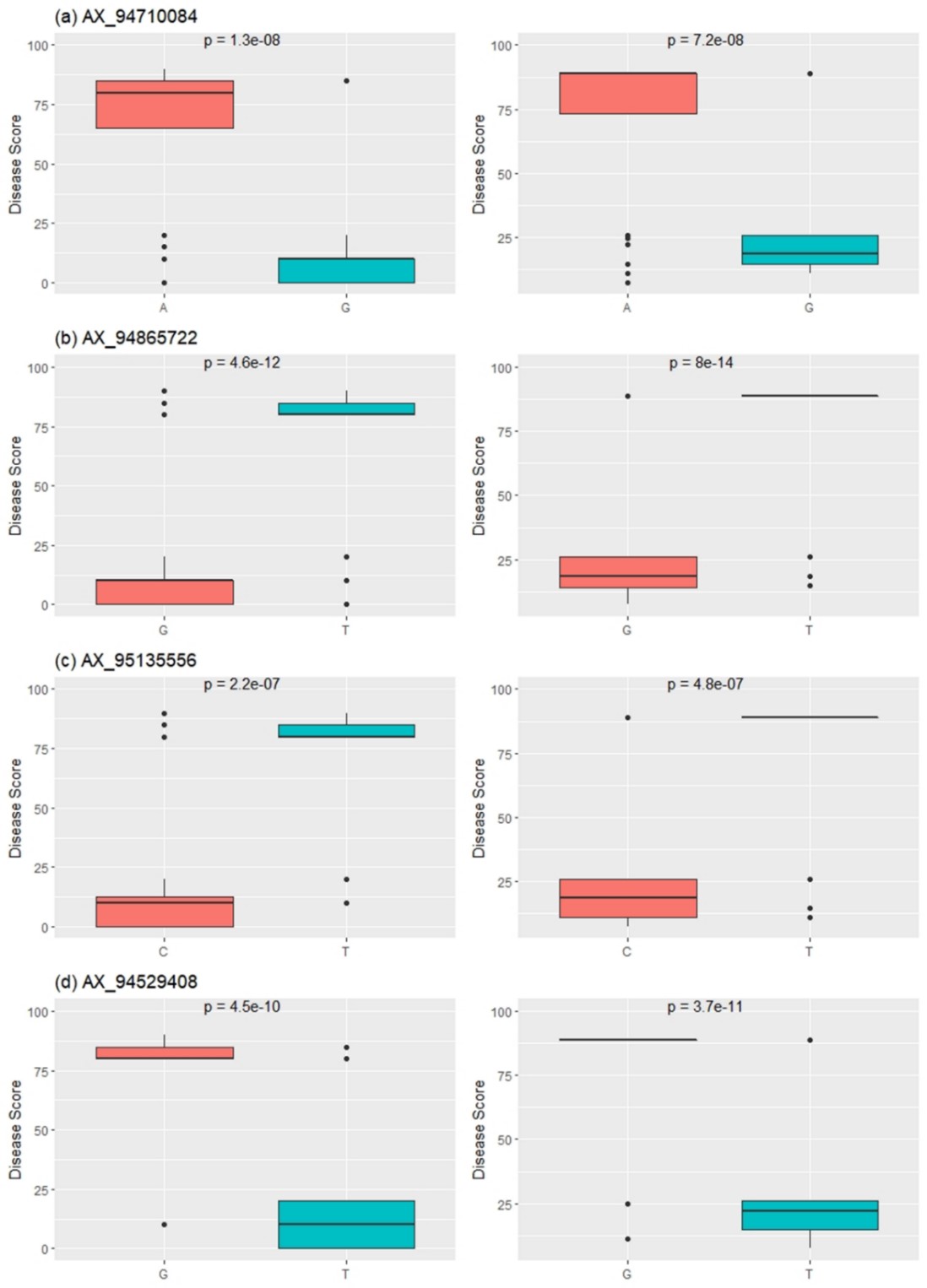 |
| --- |
| **Fig. S4** KASP validation by the effect of phenotypic variation between the two alleles of the SNPs for disease score of bread wheat; the Kruskal–Wallis test was used to determine the significant differences between the mean values of two alleles |


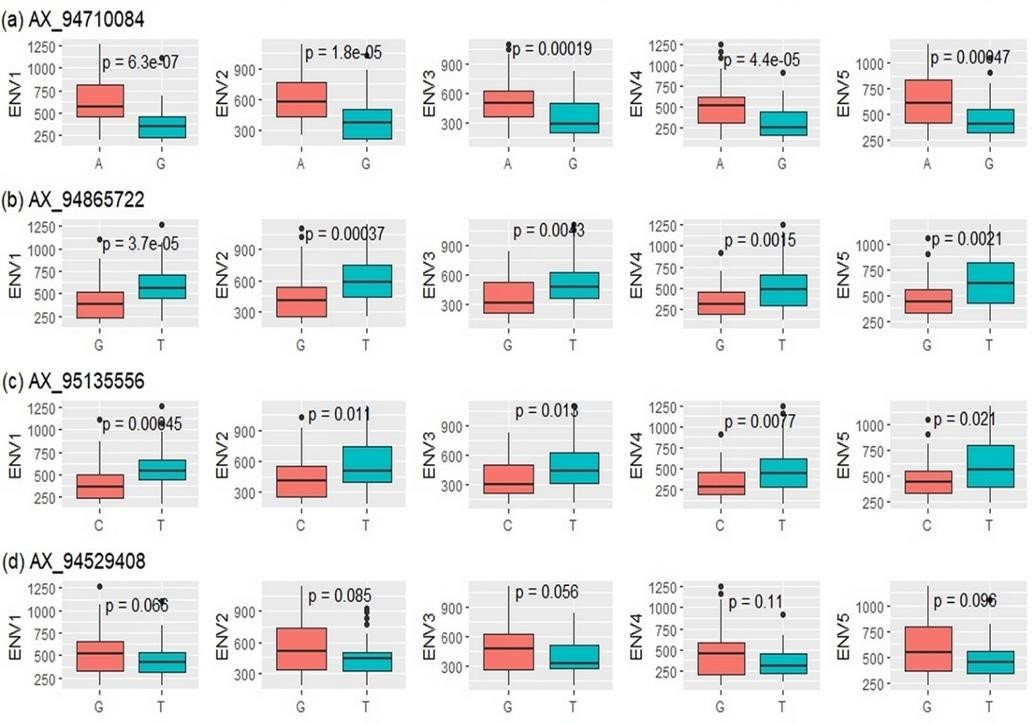


**Fig. S5** KASP validation by the effect of phenotypic variation between the two alleles of the SNPs for AUDPC of spot blotch of bread wheat; the Kruskal–Wallis test was used to determine the significant differences between the mean values of two alleles across 5 environments (Env1, Env2, Env3, Env4, Env5)


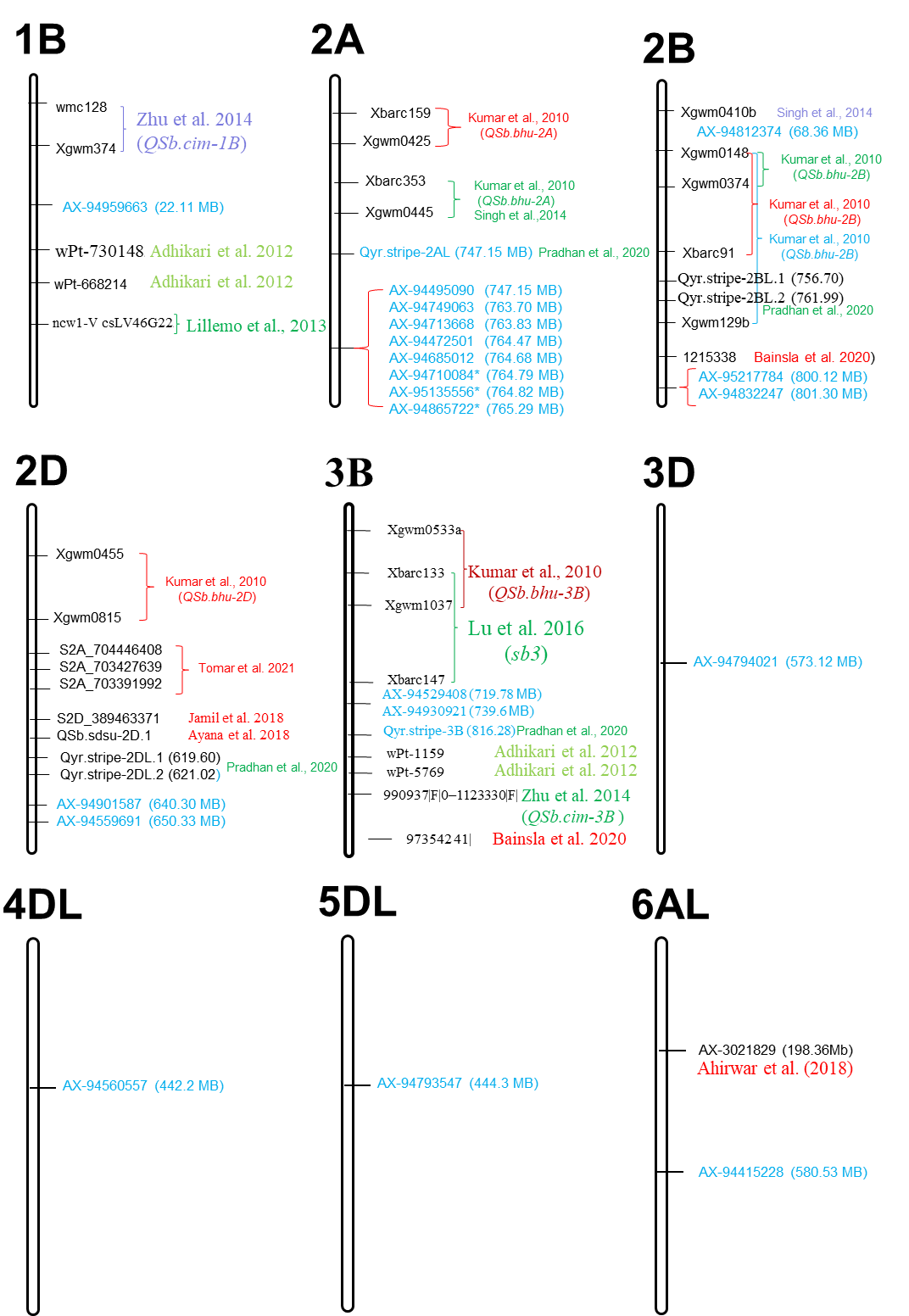


**Fig. S6** Summary of previously Identified QTL/Markers associated with spot blotch resistance correspond to Newly identified MTAs

| 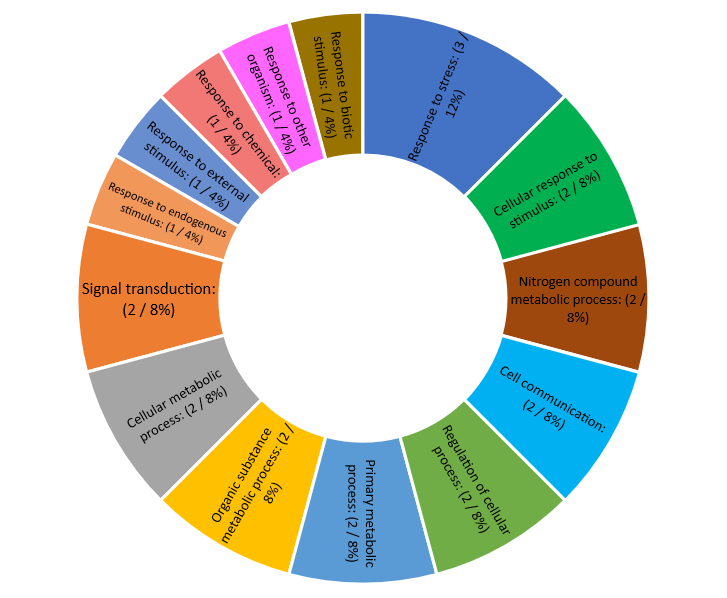  a |
| --- |
| b 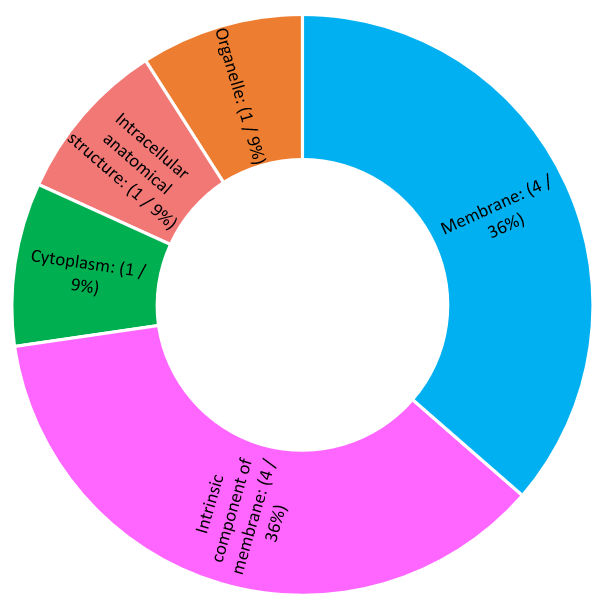 |
| c 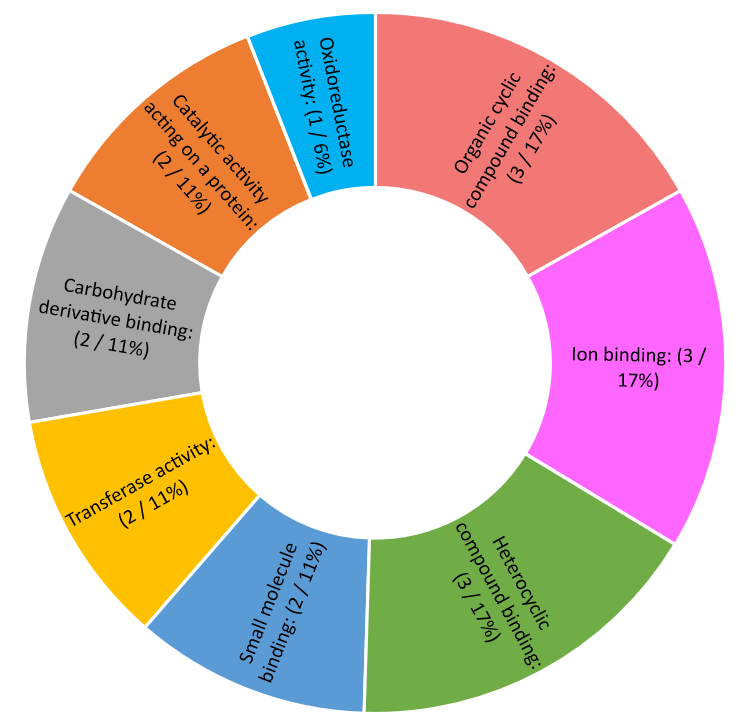 |
| **Fig. S7:** GO terms of identified candidate genes **a**) biological process **b**) cellular component **c**) molecular function. |
